# P-1010. Clinical and Economic Burden of Changing Initial Antifungal Regimens in Invasive Aspergillosis: A Hospital Perspective

**DOI:** 10.1093/ofid/ofae631.1200

**Published:** 2025-01-29

**Authors:** Barbara D Alexander, Mark Bresnik, Ruthwik Anupindi, Lia Pizzicato, Mitchell DeKoven, Belinda Lovelace, Craig I Coleman, Melissa D Johnson

**Affiliations:** Duke University School of Medicine, Durham, North Carolina; F2G, Ltd., Princeton, New Jersey; IQVIA, Falls Church, Virginia; IQVIA, Falls Church, Virginia; IQVIA, Falls Church, Virginia; F2G, Inc., Princeton, New Jersey; University of Connecticut, Storrs, Connecticut; Duke University, Durham, North Carolina

## Abstract

**Background:**

Initial antifungal (AF) regimens (AFR) for Invasive Aspergillosis (IA) are often changed due to treatment failure, intolerability, or drug-drug interactions. We compared outcomes among IA patients who did and did not change their initial IA AFR.

**Table 1.**
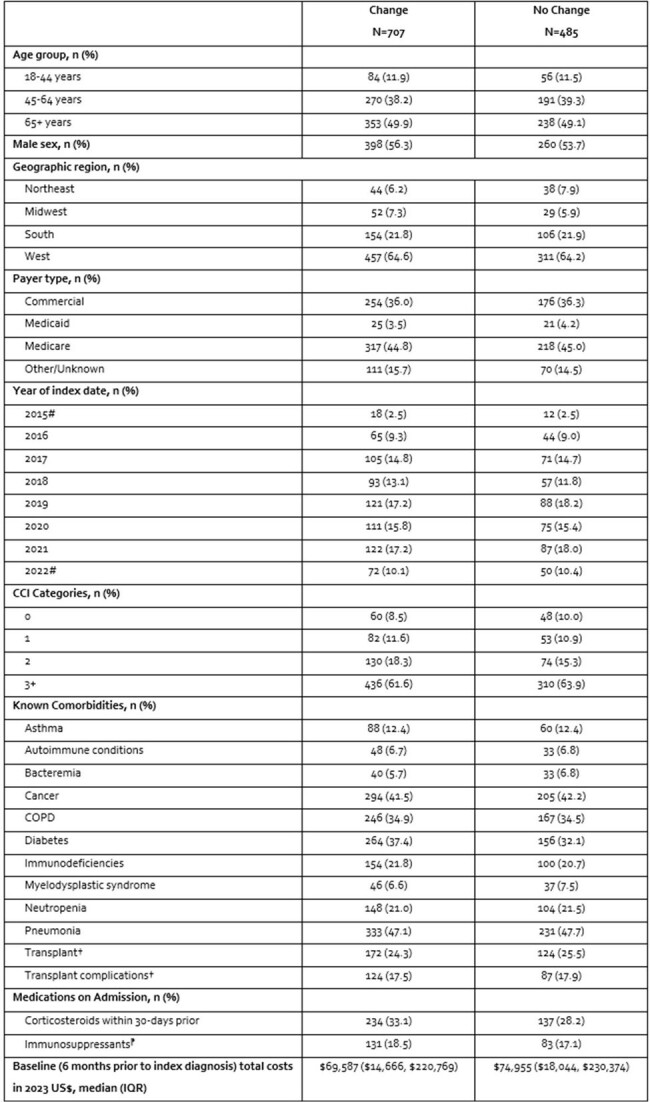
Adjusted Baseline Characteristics of Patients Who Changed or Did Not Change Their IA Antifungal Regimen* CCI=Charlson comorbidity index; COPD=chronic obstructive pulmonary disease; IA= invasive Aspergillosis; SD=standard deviation *The following covariates were used to calculate propensity scores for Inverse probability of treatment weighting: age group, geographic region, payer type, year of index, asthma, autoimmune conditions, bacteremia, COPD, pneumonia, immunodeficiencies, myelodysplastic syndrome, transplant history, immunosuppressants, cancer, neutropenia, and total healthcare costs. ||All covariates were well balanced as indicated by an SMD <0.1 except corticosteroids which was marginally imbalanced with an SMD of 0.108 †Solid organ or hematopoietic cell transplant #Partial years data available in IQVIA New Data Warehouse ⁋Azathioprine, cyclophosphamide, cyclosporine, methotrexate, mycophenolate, tacrolimus, voclosporin

**Methods:**

This retrospective cohort study identified US adults with an index hospitalization for IA (ICD-10: B44.0, B44.1, B44.7) between Oct 2015 and Nov 2022 in IQVIA’s New Data Warehouse (pharmacy, outpatient and hospital claims). Patients were placed into two cohorts based on whether they changed AFRs, defined as starting a new AFR with or without discontinuing initial therapy at any subsequent point. Inverse probability of treatment weighting minimized confounding due to baseline differences between patients who changed vs. did not change their AFR. Inpatient length of stay (LOS), all-cause costs (in 2023 US$) and all-cause mortality in IA patients who did and did not change their initial AFR were reported as medians with interquartile ranges (IQR) or frequencies and compared.

**Table 2.**
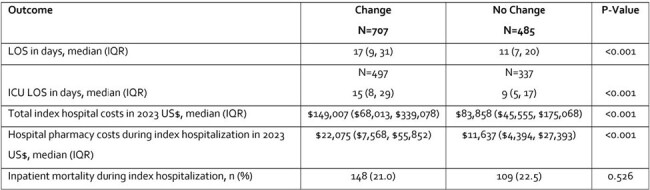
Adjusted* Outcomes of Patients Who Changed or Did Not Change Their IA Antifungal Regimen LOS=length of stay; IA= invasive Aspergillosis; ICU=intensive care unit; IQR=interquartile range; SD=standard deviation *Inverse probability of treatment weighted

**Results:**

Among 1,192 IA patients, 707 (59.3%) changed their initial AFR (**Table 1**). Most patients started therapy on an -azole (87% among those who changed vs 95% in those who did not change regimens). Duration of initial AFR for those who did and did not change their regimen was a median of 5 days (IQR=7) vs. 11 days (IQR=24), respectively. Hospital and ICU LOS were longer in the AFR change than the no change cohort (**Table 2**). Median costs associated with the index hospitalization, including pharmacy costs, were also significantly higher for those who changed AFR compared with those who did not. No statistical difference was seen in inpatient mortality during the index hospitalization between cohorts.

**Conclusion:**

In this large contemporary cohort of patients with IA, most patients changed AFRs, with many doing so within 1-2 weeks of treatment initiation. Changing regimens was associated with significantly longer hospital and ICU LOS as well as higher inpatient costs. Further study is needed to understand reasons for changing AFRs and to understand changes that might be avoided. New and improved AFs that reduce the need for regimen changes offer the opportunity to reduce the economic and clinical burden associated with changing AFRs.

**Disclosures:**

**Barbara D. Alexander, MD**, Basilea: Advisor/Consultant|F2G: Advisor/Consultant|F2G: Grant/Research Support|HealthTrackRx: Advisor/Consultant|HealthTrackRx: Board Member|Scynexis: Grant/Research Support|TFF Pharmaceuticals: Advisor/Consultant **Mark Bresnik, MD**, F2G Ltd: Employee **Ruthwik Anupindi, PhD**, F2G Ltd: Grant/Research Support|IQVIA: Employee **Lia Pizzicato, MPH**, F2G Ltd: Grant/Research Support|IQVIA: Employee **Mitchell DeKoven, PhD**, F2G Ltd: Grant/Research Support|IQVIA: Employee **Belinda Lovelace, PharmD, MS, MJ**, F2G, Inc.: Employee **Craig I. Coleman, PharmD**, F2G Ltd: Advisor/Consultant|F2G Ltd: Grant/Research Support **Melissa D. Johnson, PharmD MHS AAHIVP**, Biomeme: Licensed Technology|Scynexis, Inc: Grant/Research Support|UpToDate: Author Royalties

